# Diaqua­bis­(1*H*-imidazole-4-carboxyl­ato-κ^2^ *N* ^3^,*O*)cobalt(II)

**DOI:** 10.1107/S1600536813000330

**Published:** 2013-01-09

**Authors:** Beñat Artetxe, Leire San Felices, Aroa Pache, Santiago Reinoso, Juan M. Gutiérrez-Zorrilla

**Affiliations:** a Departamento de Química Inorgánica, Facultad de Ciencia y Tecnología, Universidad de País Vasco, UPV/EHU, PO Box 644, E-48080 Bilbao, Spain

## Abstract

The title compound, [Co(C_4_H_3_N_2_O_2_)_2_(H_2_O)_2_], contains a Co^II^ cation on a twofold rotation axis, exhibiting a distorted octa­hedral coordination geometry. The equatorial plane is formed by two *N*,*O*-bidentate 1*H*-imidazole-4-carboxyl­ate ligands and the axial positions are occupied by water mol­ecules. The crystal packing consists of a three-dimensional network stabilized by O—H⋯O and N—H⋯O hydrogen bonds, together with weak π–π inter­actions [centroid–centroid distance = 3.577 (2) Å] between the imidazole rings.

## Related literature

For the isostructural zinc(II) and cadmium(II) complexes, see: Yin *et al.* (2009 ▶); Shuai *et al.* (2011 ▶). For related homoleptic compounds, see: Kondo *et al.* (2003 ▶); Gryz *et al.* (2007 ▶); Zheng *et al.* (2011 ▶).
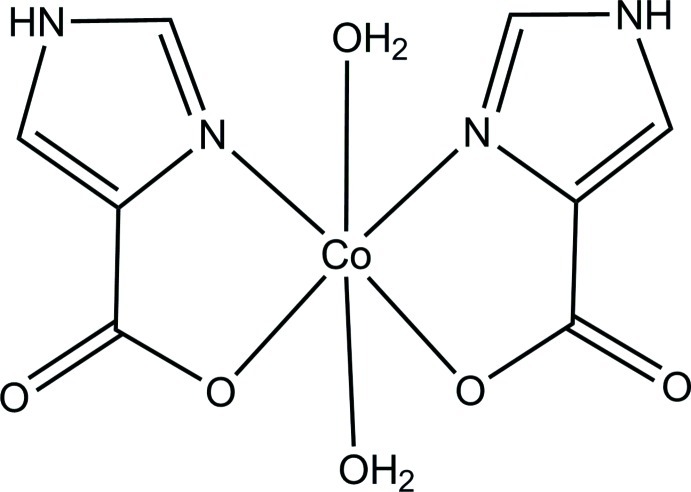


## Experimental

### 

#### Crystal data

[Co(C_4_H_3_N_2_O_2_)_2_(H_2_O)_2_]
*M* *_r_* = 317.13
Orthorhombic, 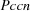

*a* = 7.1236 (16) Å
*b* = 11.6305 (2) Å
*c* = 13.5496 (4) Å
*V* = 1122.6 (3) Å^3^
*Z* = 4
Mo *K*α radiation
μ = 1.56 mm^−1^
*T* = 100 K
0.09 × 0.04 × 0.03 mm

#### Data collection

Agilent SuperNova (single source at offset) diffractometer
Absorption correction: multi-scan (*CrysAlis PRO*; Agilent, 2011 ▶) *T*_min_ = 0.947, *T*_max_ = 1.000
2396 measured reflections
1162 independent reflections
1025 reflections with *I* > 2σ(*I*)
*R*_int_ = 0.018

#### Refinement

*R*[*F* ^2^ > 2σ(*F* ^2^)] = 0.027
*wR*(*F* ^2^) = 0.063
*S* = 1.08
1162 reflections
95 parameters
2 restraints
H atoms treated by a mixture of independent and constrained refinement
Δρ_max_ = 0.32 e Å^−3^
Δρ_min_ = −0.25 e Å^−3^

### 

Data collection: *CrysAlis PRO* (Agilent, 2011 ▶); cell refinement: *CrysAlis PRO*; data reduction: *CrysAlis PRO*; program(s) used to solve structure: *SUPERFLIP* (Palatinus & Chapuis, 2007 ▶); program(s) used to refine structure: *SHELXL97* (Sheldrick, 2008 ▶); molecular graphics: *ORTEP-3 for Windows* (Farrugia, 2012 ▶); software used to prepare material for publication: *WinGX* (Farrugia, 2012 ▶) and *PLATON* (Spek, 2009 ▶).

## Supplementary Material

Crystal structure: contains datablock(s) I, global. DOI: 10.1107/S1600536813000330/zj2099sup1.cif

Structure factors: contains datablock(s) I. DOI: 10.1107/S1600536813000330/zj2099Isup2.hkl

Additional supplementary materials:  crystallographic information; 3D view; checkCIF report

## Figures and Tables

**Table:** 

Co1—N3	2.0763 (17)
Co1—O1*W*	2.1074 (15)
Co1—O1	2.1774 (14)

**Table:** 

N3—Co1—N3^i^	97.39 (9)
N3—Co1—O1*W*	98.62 (6)
N3—Co1—O1	78.47 (6)
O1*W*—Co1—O1	83.04 (6)

Symmetry code: (i) 
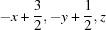
.

**Table 2:** Hydrogen-bond geometry (Å, °)

*D*—H⋯*A*	*D*—H	H⋯*A*	*D*⋯*A*	*D*—H⋯*A*
N1—H1⋯O2^ii^	0.88	1.89	2.766 (2)	172
O1*W*—H1*WA*⋯O2^iii^	0.86 (2)	1.91 (2)	2.760 (2)	171 (3)
O1*W*—H1*WB*⋯O2^iv^	0.85 (2)	1.98 (2)	2.812 (2)	167 (2)

Symmetry codes: (ii) 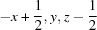; (iii) 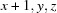; (iv) 
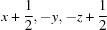
.

**Fig. 1.**
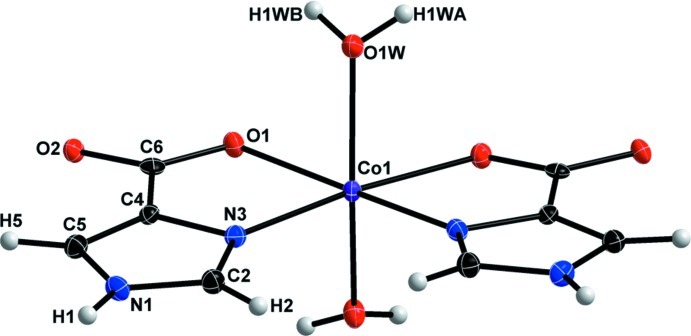
Molecular structure of [Co(C4H3N2O2)2(H2O)2] showing atom labelling for the asymmetric unit and 50% probability displacement ellipsoids.

**Fig. 2.**
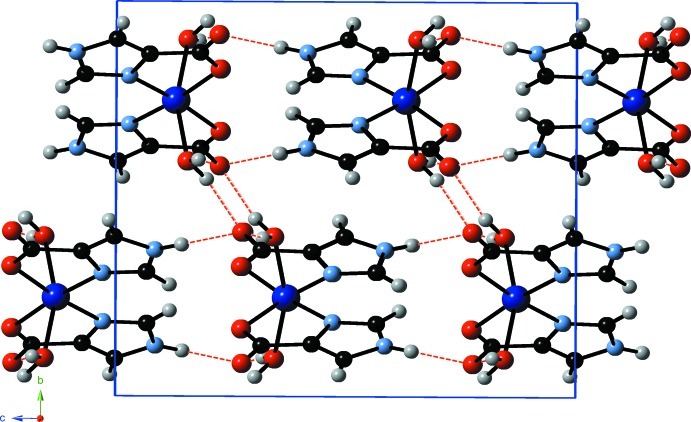
View of the crystal packing along the crystallographic a axis (hydrogen bonds represented as dashed lines).

## supplementary crystallographic information

### Comment

The title compound, [Co(C_4_H_3_N_2_O_2_)_2_(H_2_O)_2_] crystallizes in the orthorhombic crystal system, space group Pccn, and it is isostructural with the zinc and cadmium complexes previously reported by Yin *et al.* (2009) and Shuai *et al.* (2011). As expected, the Co—O and Co—N distances (Table 1) are similar to those of the Zn^II^ analogue and shorter than those of the Cd^II^ derivative. Table 2 summarizes the geometrical parameters of the O—H···O and N—H···O hydrogen bonding interactions. The centroid-to-centroid distance between interacting imidazole rings is 3.577 (2) Å.

### Experimental

To a solution of CoCl_2_.6H_2_O (12 mg, 0.05 mmol) in 15 ml of water 4-imidazole carboxylic acid (6 mg, 0.05 mmol) was added and the resulting solution was stirred for 30 min at room temperature. Prismatic red crystals were obtained by slow evaporation after several days. IR (cm^-1^): 3148 (*s*), 2934 (*s*), 1685 (*m*), 1588 (*vs*), 1555 (*vs*), 1528 (*s*), 1462 (*s*), 1406 (*vs*), 1333 (*m*), 1234 (*s*), 1177 (*m*), 1101 (*m*), 1005 (*m*), 930 (*m*), 845 (*m*), 820 (*m*), 791 (*m*), 731 (*w*), 658 (*s*), 610 (*m*), 492 (*m*).

### Refinement

All atoms except H were refined anisotropically. H atoms of the water molecule were located in a Fourier difference map and refined isotropically with O—H bond lengths restrained to 0.84 (2) Å. All imidazole H atoms were positioned geometrically and refined using a riding model with C—H = 0.93 Å, N—H = 0.86 Å and *U*_iso_(H) = 1.2*U*_eq_(C,N).

### Crystal data

**Table:**

[Co(C_4_H_3_N_2_O_2_)_2_(H_2_O)_2_]	*F*(000) = 644
*M**_r_* = 317.13	*D*_x_ = 1.876 Mg m^−^^3^
Orthorhombic, *P**c**c**n*	Mo *K*α radiation, λ = 0.71073 Å
Hall symbol: -P 2ab 2ac	Cell parameters from 1055 reflections
*a* = 7.1236 (16) Å	θ = 1.8–28.1°
*b* = 11.6305 (2) Å	µ = 1.56 mm^−^^1^
*c* = 13.5496 (4) Å	*T* = 100 K
*V* = 1122.6 (3) Å^3^	Prism, red
*Z* = 4	0.09 × 0.04 × 0.03 mm

### Data collection

**Table:**

Agilent SuperNova (single source at offset) diffractometer	1162 independent reflections
Radiation source: SuperNova (Mo) X-ray Source	1025 reflections with *I* > 2σ(*I*)
Mirror monochromator	*R*_int_ = 0.018
Detector resolution: 16.2439 pixels mm^-1^	θ_max_ = 26.5°, θ_min_ = 3.0°
ω scans	*h* = −8→7
Absorption correction: multi-scan (*CrysAlis PRO*; Agilent, 2011)	*k* = −10→14
*T*_min_ = 0.947, *T*_max_ = 1.000	*l* = −5→17
2396 measured reflections	

### Refinement

**Table:**

Refinement on *F*^2^	Primary atom site location: structure-invariant direct methods
Least-squares matrix: full	Secondary atom site location: difference Fourier map
*R*[*F*^2^ > 2σ(*F*^2^)] = 0.027	Hydrogen site location: difference Fourier map
*wR*(*F*^2^) = 0.063	H atoms treated by a mixture of independent and constrained refinement
*S* = 1.08	*w* = 1/[σ^2^(*F*_o_^2^) + (0.0201*P*)^2^ + 1.0008*P*] where *P* = (*F*_o_^2^ + 2*F*_c_^2^)/3
1162 reflections	(Δ/σ)_max_ < 0.001
95 parameters	Δρ_max_ = 0.32 e Å^−^^3^
2 restraints	Δρ_min_ = −0.25 e Å^−^^3^

### Special details

**Table:**

Experimental. CrysAlisPro, Agilent Technologies, Version 1.171.35.19 (release 27-10-2011 CrysAlis171 .NET) (compiled Oct 27 2011,15:02:11). Empirical absorption correction using spherical harmonics, implemented in SCALE3 ABSPACK scaling algorithm.
Geometry. All s.u.'s (except the s.u. in the dihedral angle between two l.s. planes) are estimated using the full covariance matrix. The cell s.u.'s are taken into account individually in the estimation of s.u.'s in distances, angles and torsion angles; correlations between s.u.'s in cell parameters are only used when they are defined by crystal symmetry. An approximate (isotropic) treatment of cell s.u.'s is used for estimating s.u.'s involving l.s. planes.
Refinement. Refinement of *F*^2^ against ALL reflections. The weighted *R*-factor *wR* and goodness of fit *S* are based on *F*^2^, conventional *R*-factors *R* are based on *F*, with *F* set to zero for negative *F*^2^. The threshold expression of *F*^2^ > 2σ(*F*^2^) is used only for calculating *R*-factors(gt) *etc*. and is not relevant to the choice of reflections for refinement. *R*-factors based on *F*^2^ are statistically about twice as large as those based on *F*, and *R*-factors based on ALL data will be even larger.

### Fractional atomic coordinates and isotropic or equivalent isotropic displacement parameters (Å^2^)

**Table:**

	*x*	*y*	*z*	*U*_iso_*/*U*_eq_	
Co1	0.75	0.25	0.13009 (3)	0.00874 (13)	
O1W	0.8972 (2)	0.09575 (13)	0.15593 (11)	0.0140 (3)	
O1	0.54278 (18)	0.17114 (12)	0.22690 (10)	0.0120 (3)	
O2	0.26134 (18)	0.08371 (12)	0.22474 (10)	0.0113 (3)	
N3	0.5553 (2)	0.18870 (15)	0.02894 (12)	0.0107 (4)	
N1	0.3584 (2)	0.12877 (15)	−0.08496 (13)	0.0125 (4)	
H1	0.3094	0.1157	−0.1435	0.015*	
C4	0.4058 (3)	0.13338 (17)	0.07356 (15)	0.0098 (4)	
C6	0.4028 (3)	0.12899 (16)	0.18291 (15)	0.0096 (4)	
C5	0.2836 (3)	0.09571 (18)	0.00325 (15)	0.0118 (4)	
H5	0.1697	0.0549	0.0136	0.014*	
C2	0.5204 (3)	0.18489 (18)	−0.06678 (15)	0.0123 (4)	
H2	0.5989	0.2173	−0.1162	0.015*	
H1WA	1.007 (3)	0.099 (3)	0.180 (2)	0.047 (9)*	
H1WB	0.847 (4)	0.050 (2)	0.1974 (17)	0.039 (9)*	

### Atomic displacement parameters (Å^2^)

**Table:**

	*U*^11^	*U*^22^	*U*^33^	*U*^12^	*U*^13^	*U*^23^
Co1	0.0080 (2)	0.0098 (2)	0.0084 (2)	−0.00165 (15)	0	0
O1W	0.0110 (8)	0.0146 (8)	0.0163 (8)	−0.0018 (6)	−0.0014 (7)	0.0039 (7)
O1	0.0102 (7)	0.0140 (7)	0.0116 (7)	−0.0016 (6)	−0.0011 (6)	−0.0008 (6)
O2	0.0087 (7)	0.0137 (7)	0.0116 (7)	−0.0007 (6)	0.0021 (6)	0.0019 (6)
N3	0.0095 (8)	0.0111 (9)	0.0115 (8)	−0.0003 (7)	0.0013 (7)	0.0009 (7)
N1	0.0130 (9)	0.0151 (9)	0.0094 (8)	−0.0007 (7)	−0.0031 (7)	−0.0005 (7)
C4	0.0101 (10)	0.0072 (10)	0.0120 (10)	0.0000 (8)	0.0007 (8)	0.0006 (8)
C6	0.0105 (10)	0.0071 (9)	0.0113 (10)	0.0041 (8)	0.0009 (8)	0.0000 (8)
C5	0.0122 (10)	0.0113 (10)	0.0118 (9)	−0.0007 (8)	0.0012 (9)	0.0001 (9)
C2	0.0121 (10)	0.0137 (11)	0.0111 (10)	−0.0008 (8)	0.0005 (8)	0.0009 (9)

### Geometric parameters (Å, º)

**Table:**

Co1—N3	2.0763 (17)	N3—C2	1.321 (3)
Co1—N3^i^	2.0763 (17)	N3—C4	1.383 (2)
Co1—O1W^i^	2.1074 (15)	N1—C2	1.349 (3)
Co1—O1W	2.1074 (15)	N1—C5	1.364 (3)
Co1—O1^i^	2.1774 (14)	N1—H1	0.88
Co1—O1	2.1774 (14)	C4—C5	1.363 (3)
O1W—H1WA	0.849 (17)	C4—C6	1.483 (3)
O1W—H1WB	0.853 (17)	C5—H5	0.95
O1—C6	1.261 (2)	C2—H2	0.95
O2—C6	1.271 (2)		
			
N3—Co1—N3^i^	97.39 (9)	C2—N3—C4	105.60 (17)
N3—Co1—O1W^i^	93.98 (6)	C2—N3—Co1	141.72 (15)
N3^i^—Co1—O1W^i^	98.62 (6)	C4—N3—Co1	112.67 (13)
N3—Co1—O1W	98.62 (6)	C2—N1—C5	108.11 (17)
N3^i^—Co1—O1W	93.98 (6)	C2—N1—H1	125.9
O1W^i^—Co1—O1W	160.87 (9)	C5—N1—H1	125.9
N3—Co1—O1^i^	174.42 (6)	C5—C4—N3	109.62 (18)
N3^i^—Co1—O1^i^	78.47 (6)	C5—C4—C6	132.70 (18)
O1W^i^—Co1—O1^i^	83.04 (6)	N3—C4—C6	117.64 (17)
O1W—Co1—O1^i^	85.47 (6)	O1—C6—O2	125.27 (18)
N3—Co1—O1	78.47 (6)	O1—C6—C4	116.60 (17)
N3^i^—Co1—O1	174.42 (6)	O2—C6—C4	118.13 (17)
O1W^i^—Co1—O1	85.47 (6)	C4—C5—N1	105.79 (17)
O1W—Co1—O1	83.04 (6)	C4—C5—H5	127.1
O1^i^—Co1—O1	105.91 (7)	N1—C5—H5	127.1
Co1—O1W—H1WA	119 (2)	N3—C2—N1	110.87 (18)
Co1—O1W—H1WB	115.7 (19)	N3—C2—H2	124.6
H1WA—O1W—H1WB	100 (3)	N1—C2—H2	124.6
C6—O1—Co1	114.52 (12)		
			
N3—Co1—O1—C6	−1.93 (13)	Co1—N3—C4—C5	179.84 (13)
N3^i^—Co1—O1—C6	−44.3 (6)	C2—N3—C4—C6	−177.35 (18)
O1W^i^—Co1—O1—C6	93.08 (13)	Co1—N3—C4—C6	1.8 (2)
O1W—Co1—O1—C6	−102.24 (13)	Co1—O1—C6—O2	−176.57 (15)
O1^i^—Co1—O1—C6	174.51 (15)	Co1—O1—C6—C4	3.4 (2)
N3^i^—Co1—N3—C2	−5.2 (2)	C5—C4—C6—O1	178.9 (2)
O1W^i^—Co1—N3—C2	94.1 (2)	N3—C4—C6—O1	−3.6 (3)
O1W—Co1—N3—C2	−100.4 (2)	C5—C4—C6—O2	−1.1 (3)
O1^i^—Co1—N3—C2	36.6 (7)	N3—C4—C6—O2	176.37 (17)
O1—Co1—N3—C2	178.6 (2)	N3—C4—C5—N1	−0.3 (2)
N3^i^—Co1—N3—C4	176.15 (16)	C6—C4—C5—N1	177.4 (2)
O1W^i^—Co1—N3—C4	−84.61 (14)	C2—N1—C5—C4	−0.2 (2)
O1W—Co1—N3—C4	80.96 (14)	C4—N3—C2—N1	−0.8 (2)
O1^i^—Co1—N3—C4	−142.1 (6)	Co1—N3—C2—N1	−179.56 (16)
O1—Co1—N3—C4	−0.06 (13)	C5—N1—C2—N3	0.7 (2)
C2—N3—C4—C5	0.7 (2)		

Symmetry code: (i) −*x*+3/2, −*y*+1/2, *z*.

### Hydrogen-bond geometry (Å, º)

**Table:**

*D*—H···*A*	*D*—H	H···*A*	*D*···*A*	*D*—H···*A*
N1—H1···O2^ii^	0.88	1.89	2.766 (2)	172
O1*W*—H1*WA*···O2^iii^	0.86 (2)	1.91 (2)	2.760 (2)	171 (3)
O1*W*—H1*WB*···O2^iv^	0.85 (2)	1.98 (2)	2.812 (2)	167 (2)

Symmetry codes: (ii) −*x*+1/2, *y*, *z*−1/2; (iii) *x*+1, *y*, *z*; (iv) *x*+1/2, −*y*, −*z*+1/2.
